# Individuality, Stability, and Variability of the Plaque Microbiome

**DOI:** 10.3389/fmicb.2016.00564

**Published:** 2016-04-22

**Authors:** Daniel R. Utter, Jessica L. Mark Welch, Gary G. Borisy

**Affiliations:** ^1^Department of Organismic and Evolutionary Biology, Harvard UniversityCambridge, MA, USA; ^2^Department of Microbiology, The Forsyth InstituteCambridge, MA, USA; ^3^Josephine Bay Paul Center for Comparative Molecular Biology and Evolution, Marine Biological LaboratoryWoods Hole, MA, USA

**Keywords:** human microbiome, 16S rRNA, community dynamics, oral microbiota, community ecology

## Abstract

Dental plaque is a bacterial biofilm composed of a characteristic set of organisms. Relatively little information from cultivation-independent, high-throughput analyses has been published on the temporal dynamics of the dental plaque microbiome. We used Minimum Entropy Decomposition, an information theory-based approach similar to oligotyping that provides single-nucleotide resolution, to analyze a previously published time series data set and investigate the dynamics of the plaque microbiome at various analytic and taxonomic levels. At both the genus and 97% Operational Taxonomic Unit (OTU) levels of resolution, the range of variation within each individual overlapped that of other individuals in the data set. When analyzed at the oligotype level, however, the overlap largely disappeared, showing that single-nucleotide resolution enables differentiation of individuals from one another without ambiguity. The overwhelming majority of the plaque community in all samples was made up of bacteria from a moderate number of plaque-typical genera, indicating that the overall community framework is shared among individuals. Each of these genera fluctuated in abundance around a stable mean that varied between individuals, with some genera having higher inter-individual variability than others. Thus, at the genus level, differences between individuals lay not in the identity of the major genera but in consistently differing proportions of these genera from mouth to mouth. However, at the oligotype level, we detected oligotype “fingerprints,” a highly individual-specific set of persistently abundant oligotypes fluctuating around a stable mean over time. For example, within the genus *Corynebacterium*, more than a dozen oligotypes were detectable in each individual, of which a different subset reached high abundance in any given person. This pattern suggests that each mouth contains a subtly different community of organisms. We also compared the Chinese plaque community characterized here to previously characterized Western plaque communities, as represented by analyses of data emerging from the Human Microbiome Project, and found no major differences between Chinese and Western supragingival plaque. In conclusion, we found the plaque microbiome to be highly individualized at the oligotype level and characterized by stability of community membership, with variability in the relative abundance of community members between individuals and over time.

## Introduction

Understanding the baseline stability or variability of the human microbiota is important for evaluating the health significance of perturbations from baseline that may occur during disease, dietary change or antibiotic treatment. A major research effort, the Human Microbiome Project (HMP, http://hmpdacc.org/) was established to provide an integrated overview of the microbial communities that share our bodies. This and other 16S rRNA gene-based studies used high throughput, large-scale cross-sectional sampling and documented a tremendous range of compositional variability between individuals and even between oral sites (Nasidze et al., 2009; Zaura et al., 2009; Segata et al., 2012; Eren et al., 2014; Xu et al., 2014). Recent studies have emphasized both that the normal human microbiota, once established, can remain stable for months or even years (Faith et al., 2013; David et al., 2014) and that the microbiota can be highly variable over short time scales (Gajer et al., 2012; Flores et al., 2014). Thus, a full understanding of the meaning of stability or variability requires connecting the measure of stability both to commonly used community analysis metrics and to a more complete analysis of the organismal composition of the community.

Most studies of stability have drawn primarily upon summary community metrics, typically involving diversity metrics and/or distance metrics to quantify and relate communities over time. One popular analytic tool, UniFrac, provides a phylogeny-based distance metric for comparison of community composition (Lozupone and Knight, 2005). Although the metric is general in nature, it is typically used with taxonomic assignments based on Ribosomal Database Project (RDP, http://rdp.cme.msu.edu/) classification at the genus level or phylotypes defined at the operational taxonomic unit (OTU) level of >97% sequence identity. These analyses provide a useful overview and allow comparison of complex data sets, but do not address the stability or variability of microbial communities at lower taxonomic levels, even when there is signal in the sequenced region of the marker gene to distinguish closely related but distinct members.

The oral cavity provides an excellent test bed for exploring questions of microbiome stability because of its accessibility and the existence of a well-curated and annotated Human Oral Microbiome Database (HOMD, http://homd.org). The HMP, in addition to gut, skin and vaginal sites, included sampling of 9 different sites in the oral cavity. Initial analysis at the genus level characterized some of the similarities and differences among the oral microbial communities (Segata et al., 2012). However, relatively few studies have investigated oral microbial dynamics and, in general, they have been analyses of community composition via summary metrics at the genus or 97% OTU level.

Several studies have emphasized the stability of microbial communities. Costello et al. (2009) analyzed oral samples, saliva and tongue dorsum, on two successive days 3 months apart and showed that variation was less within individuals than between individuals, suggesting stability, and was less over 24 h than over 3 months. Stahringer et al. (2012) analyzed saliva from 82 individuals, found no systematic change in beta diversity over 5- and 10-year intervals, and concluded that the salivary microbiome showed long-term stability. David et al. (2014) analyzed the saliva of a single individual daily for a year and found community stability over periods of months. Cameron et al. (2015) analyzed the saliva of 10 subjects at 2-month intervals for a year, found no significant community differences over the year and, therefore, concluded stability.

In contrast, other studies have emphasized the variability of microbial communities over their stability. Ding and Schloss (2014) analyzed oral HMP data at 2 or 3 time points between 30 days and 1 year. They analyzed a range of body sites and found gut and vagina to be most stable whereas the oral cavity was reported to be least stable. A study of the tongue dorsum community from two individuals with daily sampling over a year emphasized the temporal variability in the tongue dorsum community, documenting drastic shifts in relative abundance of community members at daily time scales (Caporaso et al., 2011). A detailed study by Flores et al. (2014) analyzed multiple body sites, including the tongue dorsum, of 85 subjects weekly over 3 months. Their results pointed to variability of the microbial communities and that individuals differed in the degree to which their microbiomes were variable. Thus, oral microbial dynamics have been characterized paradoxically by the seemingly contradictory qualities of stability and variability.

In an effort to deepen understanding of the oral microbiome beyond summary measures of community membership, we have evaluated microbiome composition and temporal dynamics at the species or sub-species level, using high-resolution analysis of sequence data. Recently, we used an information theory-based approach called oligotyping (Eren et al., 2013) to re-analyze the HMP 16S rRNA gene sequence data of the entire oral microbiome at the single-nucleotide level. We compared the observed sequences to the curated HOMD (Dewhirst et al., 2010) and found that most sequences were exact or near-exact matches to known oral taxa. Some oral microbes differed from one another by only a few nucleotides in the sequenced region of the 16S rRNA gene, but the single-nucleotide resolution of oligotyping made it possible to distinguish them from one another. Some of these closely related sequences matched the same reference taxon in HOMD but were abundant at different oral sites. We interpret these distinctive distributions as demonstrating a level of ecological and functional diversity not previously recognized (Eren et al., 2014). We then applied oligotyping to the tongue dorsum by re-analyzing the Caporaso et al. (2011) data set. We identified a persistent core tongue dorsum microbiome but with rapidly (daily) fluctuating proportions of the characteristic taxa (Mark Welch et al., 2014): Some oligotypes were stable for months but underwent abrupt transitions to alternate oligotypes within days. However, it remained an open question whether this finding was specific to the tongue dorsum community, and the closely related salivary microbiome, or whether other oral communities exhibited similar community dynamics.

Recently, Jiang et al. (2015) published a high-quality data set of the plaque microbiome of eight different Chinese individuals over a period of 3 months. Their objective was to define a “dynamic core microbiome,” the set of taxa present at all time points in all individuals. Consequently, they pooled the data from all eight individuals at each time point. However, the same data, when kept un-pooled, provides an opportunity to investigate questions of stability and variability of the plaque microbiome within individuals, as well as to compare these samples to those of the Human Microbiome Project, collected in the United States. Here we re-analyze this high-quality plaque time series data with single-nucleotide resolution. Our results provide evidence for a unique community “fingerprint” in the plaque of each individual, consisting of a set of organisms and their relative abundances that fluctuate around a stable mean within each individual over the months-long sampling period.

## Methods

### Sample collection and sequence acquisition

This is a re-analysis of existing sequence data; procedures for informed consent, institutional approval, and sample collection and sequencing are described in the original publication (Jiang et al., 2015). Briefly, Jiang et al. collected supragingival plaque samples from eight healthy, 25- to 28-year-old Chinese subjects at eight time points: Day 0, Day 1, Day 3, Week 1, Week 2, Week 3, Month 1, and Month 3 for a total of 64 samples. In their study, the V4-V5 hypervariable region of the 16S rRNA gene was sequenced using Roche 454 GS-FLX pyrosequencing; sequence data was stored in the NCBI sequence read archive (SRA) under SRA accession number SRP049987 (http://www.ncbi.nlm.nih.gov/Traces/study/?acc=SRP049987; Jiang et al., 2015). The total data available from NCBI consisted of 359,565 reads, with an average of 5618 sequences per sample (*SD* = 923.8). To eliminate the artificial length variation among reads introduced by the original quality trimming, we re-trimmed each read to 336 nucleotides and removed the reads that were shorter, which reduced the size of the data set by less than 10%. These reads were then aligned against the GreenGenes reference alignment (McDonald et al., 2011; greengenes.lbl.gov/Download/OTUs/gg_otus_6oct2010/rep_set/gg_97_otus_6oct2010_aligned.fasta) using PyNAST version 0.1 (biocore.github.io/pynast/) (Caporaso et al., 2010), and we removed positions from the resulting alignment that consisted only of gap characters. The final data set contained a total of 301,657 reads.

We also downloaded data from Eren et al. (2014), which was part of an oligotyping re-analysis of data from The Human Microbiome Project Consortium (2012). The downloaded data consisted of supplemental data sets, Dataset_S01 (V1-V3 oligotypes) and Dataset_S02 (V3-V5 oligotypes). No re-analysis was done on this data; it was only collapsed to the genus level and reformatted for comparison to both the original Jiang et al. (2015) work and our re-analysis of it.

### Minimum entropy decomposition and taxon assignment

Minimum Entropy Decomposition (MED; Eren et al., 2015) is an automated data analysis algorithm that operates on the same principle as oligotyping (Eren et al., 2013). MED uses high-entropy nucleotide positions to iteratively partition a given collection of reads into *de novo* bins we will refer to as oligotypes. We used the MED pipeline, version 2.0, (Eren et al., 2015) to decompose the data set. The “minimum substantive abundance” criterion (−M) was set to 60, and the “maximum variation allowed” criterion (−V) was set to 3. Minimum entropy decomposition of 301,657 reads generated 333 oligotypes, retaining a total of 227,991 sequences. Of the 73,666 reads removed, 58,591 reads were removed due to the minimum substantive abundance criterion and 15,075 reads were removed due to the maximum variation allowed criterion. A visual breakdown of the taxonomy of reads lost in each step of the process, from alignment to final output, is presented in Supplementary Image 1. Taxonomy was then assigned to each of the 333 oligotypes by querying the representative sequence of each oligotype against the Human Oral Microbiome Database (HOMD) RefSeq v.13.2 (www.homd.org, Dewhirst et al., 2010) using the Global Alignment Search Tool (GAST; Huse et al., 2008). Taxonomic data and abundance by oligotype are presented in Supplementary Data Sheet 1.

### Data processing and figure creation

We used R (version 3.2.2; R Core Team, 2015) for all post-MED data analysis. We used the *metaMDS* function in the vegan package (Oksanen et al., 2013) to generate the MDS analysis shown in **Figures 2**, **3**. All figures were created with the *ggplot* function in the ggplot2 package (Wickham, 2009). After generation in R, figures were cleaned and processed for final publication with Inkscape (version 0.91, http://inkscape.org/).

## Results

### Analysis of plaque 16S rRNA gene sequencing data with single-nucleotide resolution

We used Minimum Entropy Decomposition (MED; Eren et al., 2015) to re-analyze the time series data generated from the supragingival plaque samples of eight individuals at eight time points over 3 months (Jiang et al., 2015). This data set was of interest because it presented an opportunity to analyze the temporal dynamics of the plaque microbiome at single-nucleotide resolution.

**Figure 1:**
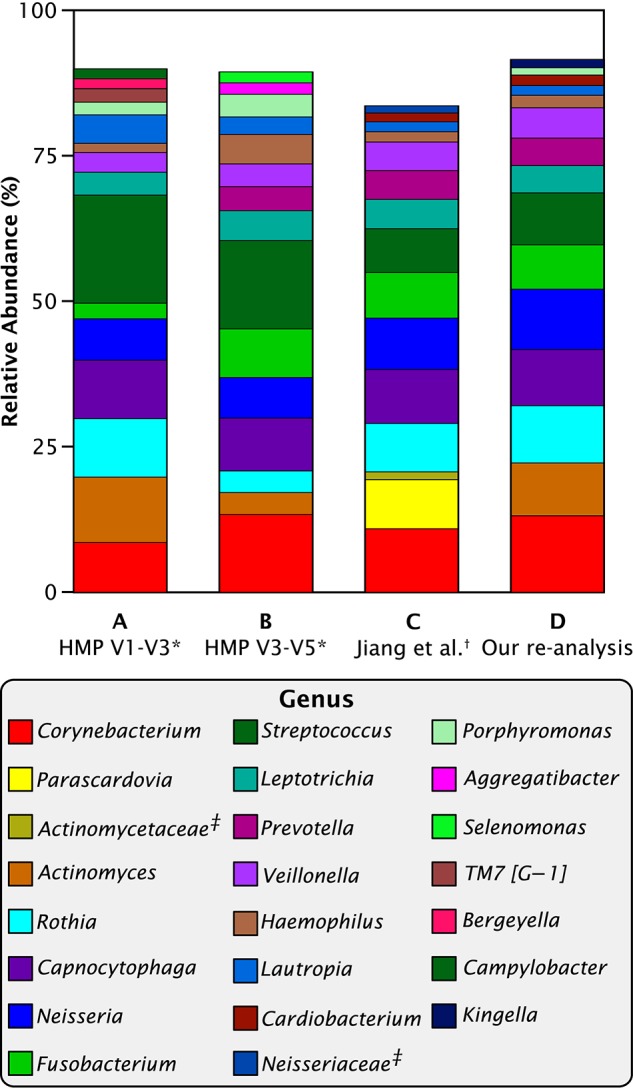
**Abundant genera in Chinese and Western plaque**. Mean relative abundances of the most abundant 15 taxa in HMP and Jiang et al. data. **(A)** ^*^Eren et al. (2014) re-analysis of Human Microbiome Project V1-V3 supragingival plaque data; **(B)** ^*^Eren et al. (2014) re-analysis of HMP V3-V5 data; **(C)** ^†^data from supplemental table S2 of Jiang et al. (2015); **(D)** our re-analysis of Jiang et al. data. ^‡^Sequences in table S2 of Jiang et al. (2015) that were labeled to the family level only.

However, before pursuing the question of microbiome dynamics, we had to address a striking difference between the genus-level results of Jiang et al. (2015) and those emerging from the Human Microbiome Project. Jiang and co-authors reported a high percentage of an unusual taxon, *Parascardovia*, and a low percentage of *Actinomyces* in contrast to the Human Microbiome data as re-analyzed by Eren et al. (2014), which showed no *Parascardovia* and substantial amounts of *Actinomyces* (Figure 1). Otherwise, the two studies were in general agreement. We asked whether the disparity could have arisen from genetic, cultural, or environmental differences between the two populations or from technical causes such as method of informatics analysis or sequencing strategy. The HMP data was collected and analyzed over two different regions of the 16S RNA gene, V1-V3 and V3-V5. Although these two regions give slightly different abundance values for plaque genera, their results were similar and neither contained *Parascardovia*. The Jiang et al. study sequenced the V4-V5 region, which overlaps the V3-V5 region of the HMP study. Therefore, the location of the sequenced region was not a sufficient explanation for the disparity.

When we re-analyzed the Jiang et al. sequence data with the MED pipeline and then categorized the resulting oligotypes into genera, the disparity disappeared (Figure 1). In our re-analysis of the Jiang et al. data, no sequences were identified as *Parascardovia* and the abundance of *Actinomyces* was within the range of variation of the HMP data. Consequently, we conclude that the disparity between the two data sets is likely to be methodological and does not reflect any genetic, cultural, or environmental difference between the two populations. The genera *Parascardovia* and *Actinomyces* are in the same phylum, Actinobacteria, and it is possible that sequences representing *Actinomyces* were misclassified as *Parascardovia* in Jiang et al.'s original analysis. We evaluated possible mechanisms of error in classification (Supplementary Data Sheet 2) but were unable to identify the exact source of the misclassification. Nevertheless, the salient point for this study is that when the sequence data was processed through the MED pipeline, the disparity between the Jiang et al. and the HMP results disappeared.

### Composition of Chinese and Western supragingival plaque is broadly similar at the genus level

Next, we wanted to understand how Chinese plaque composition compared to Western plaque, as represented by the HMP data. As a basis for comparison of the Chinese and the two HMP data sets, we used the relative abundances of the 40 genera shared between all three data sets. These shared genera made up the vast majority of all the sequence reads: non-shared taxa comprised only 3.2% of the V1-V3 and 2.2% of the V3-V5 re-analyzed HMP data (Eren et al., 2014), and 0.9% of the re-analyzed Jiang et al. data. Differential presence or absence of low-abundance, non-shared genera may result from technical differences in experimental design and we reasoned that eliminating them from the analysis would allow for a more parsimonious comparison across studies. Supplementary Data Sheet 3 provides a breakdown of mean relative abundances by genus in each data set.

**Figure 2:**
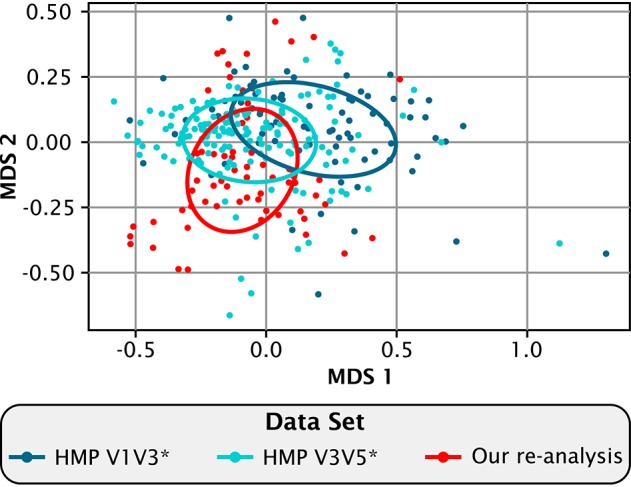
**Compositional similarity between Chinese and Western plaque**. Multidimensional scaling plot showing the relatedness among the re-analyzed ^*^(Eren et al., 2014) HMP V1-V3 (dark blue), and HMP V3-V5 data sets (light blue), and our re-analysis of the Jiang et al. (2015) data (red). Each dot represents an individual sample. Sample distances were calculated by the Bray-Curtis dissimilarity index using the relative abundances of the 40 genera shared between all three data sets. Ellipses bound the covariance of each data set and are centered on the mean of that set.

Comparing the re-analyzed data from Jiang et al. to our analysis of the HMP data revealed that Chinese and American plaque have a similar overall composition when viewed at the genus level (Figure 2). On a multi-dimensional scaling (MDS) plot of Bray-Curtis distances, covariance ellipses representing the HMP data from the V1-V3 (blue) and the V3-V5 (cyan) regions of the 16S rRNA gene overlap but do not entirely coincide, showing that similar but not identical communities are recovered when the same DNA sample is analyzed using two different regions of the marker gene. The re-analyzed Jiang et al. data in Figure 2 (red) also overlaps substantially with the HMP data, showing that within the range of experimental variation there is no detectable difference at the genus level between Chinese and Western plaque.

### Intra-individual plaque variability is less than inter-individual variability

**Figure 3:**
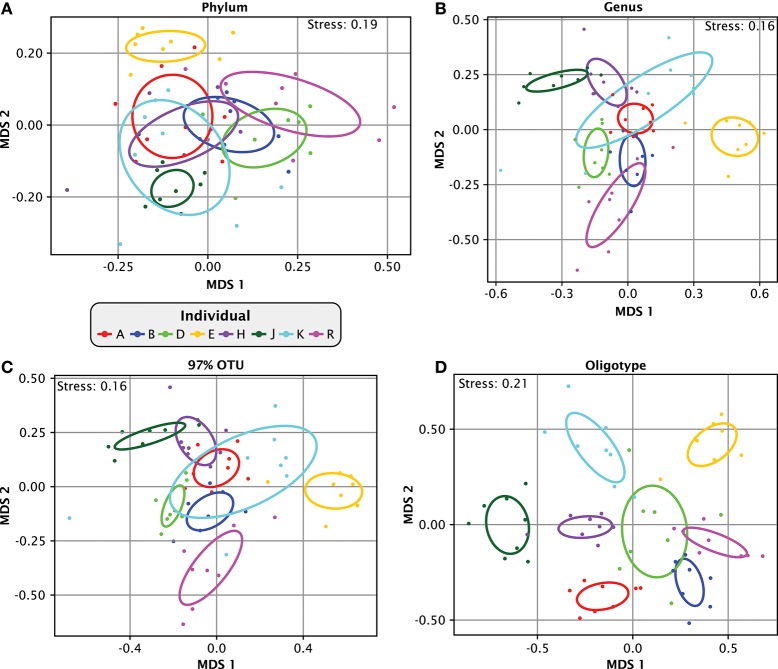
**Apparent similarity of samples depends on taxonomic resolution**. Multidimensional scaling (MDS) plots of relatedness of samples from different individuals. Bray-Curtis dissimilarity index was calculated based on relative abundances in each individual of all **(A)** phyla, **(B)** genera, **(C)** OTUs at 97% identity and **(D)** oligotypes. For **(C)**, 97% OTUs were created by clustering the oligotype representative sequences using the centroid clustering method according to the Bray-Curtis distances. From this clustered matrix we binned together all oligotypes that were at least 97% similar to any oligotype in the same bin but were not already included in another bin. The ellipses mark the covariance of each individual data set; they are centered on the mean of each individual and colored by individual.

The time series information provided by the Jiang et al. data set provides an unusual opportunity to analyze intra-individual variation in plaque over time relative to inter-individual variation within the same study. However, to evaluate intra-individual temporal dynamics, we needed to establish a level of analytic resolution sufficient to clearly distinguish the plaque microbiota of individuals. Figure 3 shows multidimensional scaling (MDS) plots at various levels of analytic resolution. Analyses for all samples were carried out based on relative abundances of all taxa. At the phylum level (Figure 3A), the plots for individuals largely overlapped one another. Not surprisingly, the overlap was greatly reduced at the genus level (Figure 3B). Close inspection of Figure 3B shows that samples from most of the individuals overlapped with those from only two or three neighbors, and samples from one individual (E, colored orange) were distinct from all the other individuals. Similar results were obtained at the operational taxonomic unit (OTU) level of 97% sequence identity (Figure 3C). Thus, at both the genus and 97%-OTU levels of resolution, individuals showed variation in plaque composition from sample to sample, with the variation for each individual, in most cases, ranging over a relatively small fraction of the total range of variability of the population. However, overlap among individuals still occurred.

Increasing the resolution beyond the genus or 97%-OTU level revealed far more dramatic distinctions between individuals. When the same data was plotted at the oligotype level (Figure 3D), the covariance overlap between individuals diminished drastically and individuals occupied largely non-overlapping regions of the plot. The stress function of the MDS plot in Figure 3D has a relatively high value of 0.2, but the topology was consistent in 49 out of 50 trials (Supplementary Image 3). This result shows that the single-nucleotide-level resolution of oligotyping reveals individual-level differences that are less apparent at the genus or 97%-OTU level. Taken together, our analysis demonstrates that the level of similarity observed within and between individuals is dependent on the taxonomic resolution at which the data is analyzed and that single-nucleotide resolution enables differentiation of individuals from one another without ambiguity.

### Moving from summary metrics to understanding community composition

Summary metrics such as MDS plots based on the Bray-Curtis dissimilarity index provide a measure of the degree of overall difference between microbial communities. However, like any summary metric, they inevitably obscure underlying key information. For example, the plots *per se* do not distinguish between differences arising from the presence of the same taxa in differing proportions or from the presence of different taxa. This distinction is of biological importance because it reflects upon the fundamental membership of microbial communities. Understanding the nature of the differences in the plaque community between individuals therefore requires moving from summary metrics to a more detailed analysis of the data itself, specifically the community composition of individual samples.

**Figure 4:**
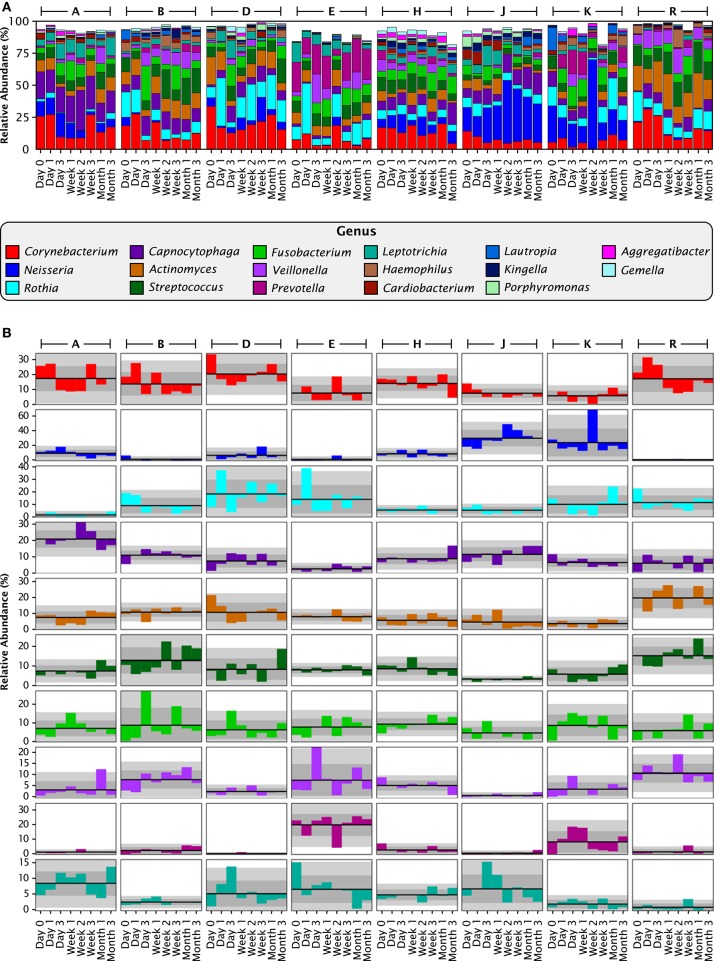
**Stable differences between individuals at the genus level. (A)** Relative abundances for each individual at each time point, for all 17 genera with greater than 1% mean relative abundance over all 64 samples. Together these genera compose 93.8% of the data set. **(B)** Anomaly from the mean relative abundance for each sample from each individual. The mean relative abundance for an individual is marked by the dark line, and one and two standard deviations by the dark and light gray fields, respectively. Columns represent individuals, and rows represent genera, with colors as in **(A)**.

Deconstructing each sample by analyzing the relative abundance of taxa revealed that most of the plaque community was made up of bacteria from a moderate number of plaque-typical genera. A set of 17 genera was present in every individual at almost every time point and collectively made up between 80 and 99% of each plaque sample (Figure 4A). Thus, at the genus level of taxonomic resolution, the bulk of the plaque community was composed of a consistent set of taxa in all individuals, supporting the view of a core temporal plaque microbiome at the genus level.

To assess the stability of taxonomic composition and its consistency across individuals, we used the straightforward metrics of mean, standard deviation, and coefficient of variation. For the 8 samples from each individual, we calculated the mean abundance and its standard deviation for each genus; deviations from the mean are plotted in Figure 4B for the 10 genera with the highest mean relative abundances. For most taxa, the fluctuations were within 2 standard deviations from the mean, as expected because the mean and standard deviation are themselves calculated from the eight data points. More interestingly, the mean relative abundance for these major genera was stable over 3 months within each individual. Taxa exhibited shifts in relative abundance over time within an individual, but the shifts were generally fluctuations around an individual mean rather than displaying an increasing or decreasing trend (Figure 4B). Further, the mean was relatively constant across individuals for some genera, but in other genera differed substantially from individual to individual. This distinction can be observed visually in Figure 4B, as illustrated by the even distribution of *Fusobacterium* (shown in bright green) in contrast to the variability of *Prevotella* (maroon) and *Neisseria* (dark blue). The distinction is also evident in the coefficient of variation. Five relatively constant genera, *Streptococcus, Corynebacterium, Capnocytophaga, Fusobacterium*, and *Actinomyces*, each had a coefficient of variation between 58 and 68% over all 64 samples (Table 1) and between 40 and 66% on average within the 8 samples from a single individual (Table 1). The more variable genera, such as *Neisseria* and *Prevotella*, had higher coefficients of variation, 126 and 155% respectively across all 64 samples, and 90 and 97% on average within each individual. Some fluctuations were large as a fraction of the overall community, such as the shift of *Prevotella* in individual E from 25 to 4% and back to 21% of the community in consecutive samples, or the shift in *Neisseria* in individual D from 3 to 19% and back to 4% (Figure 4A). Interestingly, the two individuals in whom the aerobic *Neisseria* was most abundant, individuals J and K, were also the individuals with the lowest fraction of the facultatively aerobic *Streptococcus* (Figure 4A). In summary, differences between individuals, as assayed at the genus level, lay not in the identity of the major genera but in consistently differing proportions of these genera from mouth to mouth.

**Table 1:** **Temporal stability varies between genera**.

	**A**		**B**		**D**		**E**		**H**		**J**		**K**		**R**		**Overall**		
	**M**	**CV**	**M**	**CV**	**M**	**CV**	**M**	**CV**	**M**	**CV**	**M**	**CV**	**M**	**CV**	**M**	**CV**	**M**	**CV**	**Mean CV**
Cor	17.4	48	13.9	56	20.4	34	7.8	70	14.3	35	7.7	41	6.1	58	17.2	50	13.1	60	49
Nei	9.5	54	1.3	169	6.8	80	1.2	190	8.8	46	30.2	36	24.2	78	0.8	71	10.3	126	90
Rot	1.9	85	9.4	65	18.8	60	14.4	77	5.9	35	5.8	46	10.4	71	11.8	48	9.8	84	61
Cap	20.9	25	11.3	25	7.7	52	2.9	64	9.1	37	11.8	36	6.8	40	6.4	68	9.6	64	43
Act	7.7	49	11.1	26	10.9	54	8.2	30	6	58	4.8	87	3.9	57	19.8	32	9	68	49
Str	7.7	38	13	51	8.5	63	8.3	22	8.8	35	3.5	25	6.1	57	15.3	30	8.9	58	40
Fus	7.4	57	9.1	101	6.6	76	8.1	56	9.6	34	4.9	66	8.9	63	6.1	75	7.6	68	66
Vei	3.3	120	7.9	51	2.5	62	7.6	94	5.2	44	0.7	118	3.6	87	10.7	39	5.2	92	77
Pre	1.4	66	2.5	94	0.4	120	19.9	38	2.8	82	0.6	167	8.4	88	1.6	120	4.7	155	97
Lep	8.5	46	2.6	39	5.2	78	6.7	66	4.9	36	6.8	66	1.9	75	0.9	133	4.7	83	67

*For each genus (row) the mean relative abundance (M) and coefficient of variation (CV) are shown. The CV is presented as a percentage. In columns A, B, D, E, H, J, K, and R, mean and CV are calculated across the 8 samples from the respective volunteer. The mean and CV columns under the heading “Overall” were calculated from all 64 samples. The Mean CV column is the mean of all the CVs calculated from the individuals, i.e., the mean of [CV(A) …CV(R)]. Genus abbreviations: Cor, Corynebacterium; Nei, Neisseria; Rot, Rothia; Cap, Capnocytophaga; Act, Actinomyces; Str, Streptococcus; Fus, Fusobacterium; Vei, Veillonella; Pre, Prevotella; Lep, Leptotrichia*.

**Figure 5:**
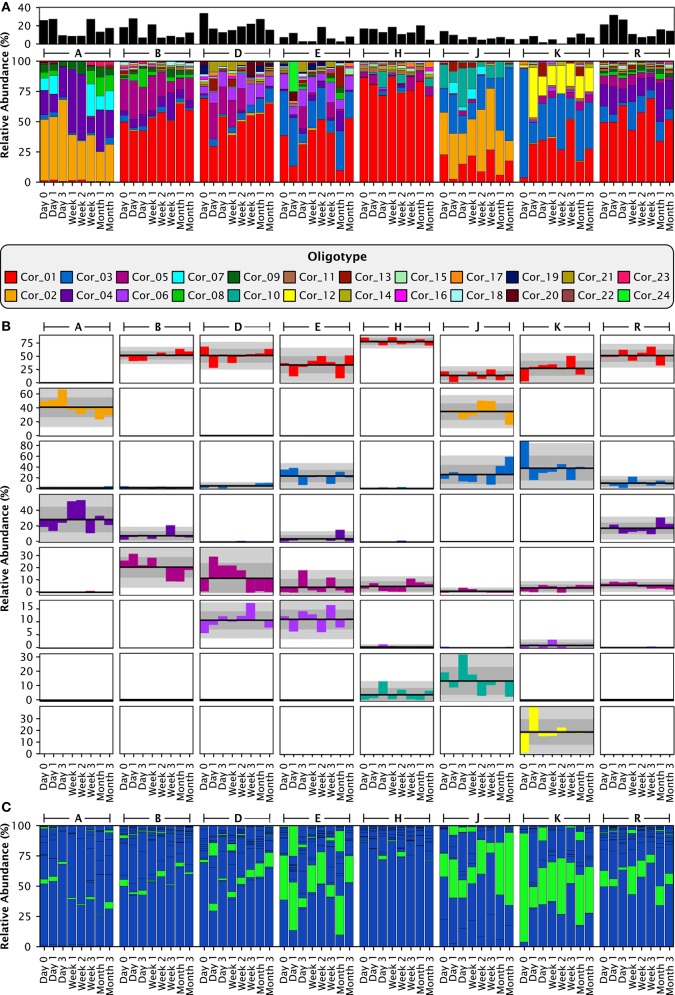
**Stable differences between individuals are clear at the oligotype level**. **(A)** Relative abundances of the 24 *Corynebacterium* oligotypes. The smaller, black stackbar shows the *Corynebacterium* abundance relative to all taxa in the sample, while the larger, colored stackbar shows the abundance of each *Corynebacterium* oligotype relative to the total abundance of *Corynebacterium* in each sample. **(B)** Anomaly from the mean relative abundance for each sample from each individual. The mean relative abundance for an individual is marked by the dark line, and one and two standard deviations by the dark and light gray fields, respectively. Columns represent individuals and rows represent oligotypes, colored as in **(A)**. **(C)** Exactly the same data and organization as **(A)**, but colored by species instead of by oligotype. *C. matruchotii* oligotypes are colored blue; *C. durum*, green.

Analysis with single-nucleotide resolution, however, revealed that within certain genera, each individual carried a distinctive set of organisms, as revealed by a distinctive pattern of oligotypes distinguishable by at least one nucleotide in the sequenced portion of their 16S rRNA gene. To illustrate this point, we decomposed *Corynebacterium*, the most abundant genus, into its 24 distinct oligotypes (Figure 5A). Between 4 and 19 *Corynebacterium* oligotypes were detectable in a single time point and between 15 and 24 unique oligotypes were detectable in each individual. However, only a handful of these oligotypes reached high abundance in each individual, and these oligotypes tended to maintain that high abundance within the individual over time. Unlike the genus-level analysis, visual comparison of individuals at the oligotype level (e.g., individuals A and H; Figure 5A) showed that individuals had strikingly different oligotype profiles, defining “profiles” as the combination of community membership and relative abundance. Figure 5B displays the anomaly from the mean for eight *Corynebacterium* oligotypes that were of high relative abundance (mean >10% of the *Corynebacterium*) in any individual. As in the genus-level analysis, the abundance of taxa fluctuated about a mean that was stable for each individual. A stable mean within an individual, but sharp differences between individuals, was confirmed by examination of the coefficient of variation: for the 8 *Corynebacterium* oligotypes of high relative abundance, the mean within-mouth coefficient of variation was 45% in mouths in which that oligotype was >10% of the *Corynebacterium* but was much higher, 173%, across all samples from all individuals, reflecting the consistent abundance of these oligotypes in some mouths and their near-absence in others (Table 2).

**Table 2:** **Oligotypes are stably abundant within an individual but not between individuals**.

	**A**		**B**		**D**		**E**		**H**		**J**		**K**		**R**		**Overall**	
	**M**	**CV**	**M**	**CV**	**M**	**CV**	**M**	**CV**	**M**	**CV**	**M**	**CV**	**M**	**CV**	**M**	**CV**	**M**	**CV**
Cor_01	1.4	42	52.7	**15**	52.2	**25**	35.1	**46**	78.6	**8**	15.3	**56**	28.7	**49**	52.2	**21**	39.5	64
Cor_02	41.5	**34**	0.4	138	1.1	70	0.6	124	0.2	124	35.4	**33**	0.5	98	0	283	9.9	178
Cor_03	2.6	68	2.8	64	6.2	59	24.6	**47**	1.2	120	27.4	**63**	39	**59**	11.3	**58**	14.4	119
Cor_04	29.2	**55**	8.6	67	0.4	173	4.6	107	0.3	264					18.2	**40**	7.7	157
Cor_05	0.3	194	20.8	**40**	11.8	**104**	4.4	158	5.1	81	1.1	141	3.8	74	6	45	6.7	129
Cor_06	0	186	0.1	200	10.8	**32**	11.1	**34**	0.5	108	0.2	186	1.1	102	0.2	119	3	166
Cor_10							0	283	3.7	130	13.4	**72**	0.1	283			2.2	266
Cor_12							0	283	0.1	217			19.4	**56**	0.1	200	2.4	303
Mean of “Overall CV”																		173
Mean of abundant CVs																		45

*Mean relative abundance (M) and coefficient of variation (CV) are shown for each Corynebacterium oligotype that comprises a mean of at least 10% of the total Corynebacterium reads in any individual. Mean and CV values are left blank when the oligotype was not detected in any of the 8 samples in that individual. The CV is presented as a percentage. Bolded values indicate CVs for oligotypes with a mean relative abundance of at least ≥10% of the total Corynebacterium reads in that individual. Table headings A, B, D, E, H, J, K, R represent the individual whom the statistics below represent. The mean and CV columns under the heading entitled “Overall” were calculated based the oligotype relative abundances from all 64 samples*.

Similar individual-characteristic results were obtained for most other genera (Supplementary Image 4). Oligotype abundance was highly dynamic, but as with *Corynebacterium*, the oligotypes fluctuated around a stable mean (Supplementary Images 4, 5). However, a few oligotypes, for example oligotypes of *Streptococcus* in individuals J and R, *Rothia* in individual B, and *Neisseria* in individual J, broke this generality by showing a transition from low abundance to high abundance, or vice versa (Supplementary Image 5). We may nevertheless conclude that the oligotype-level composition of the microbiota of plaque is distinctive to individuals. When viewed together, oligotype profiles across all genera provided a unique oligotype “fingerprint” for each individual.

Since species designations are the accepted standard within the oral microbiome community, it is important to relate the oligotype patterns to a species-level analysis. Of the 24 *Corynebacterium* oligotypes, 21 represent the species *C. matruchotii* and the remaining 3 oligotypes represent *C. durum* (Figure 5C). When individuals were analyzed at species level, all individuals in the data set showed the same two *Corynebacterium* species. For example, comparison of individuals A and H, who have completely distinct *Corynebacterium* compositions at the oligotype level, as shown in Figure 5A, showed indistinguishable compositions at the species level with *C. matruchotii* the dominant species and *C. durum* a minor species (Figure 5C, Supplementary Data Sheet 1). Thus, some of the distinctions in microbiota between individuals that were so visible at the oligotype level were obscured even at the species level.

The degree to which oligotypes of the 16S ribosomal RNA gene provided better-than-species-level resolution was dependent on the genus. The genus *Capnocytophaga* resembled *Corynebacterium* in having an abundance of oligotypes resulting in a highly distinctive oligotype fingerprint for each individual, even for individuals whose species-level composition was similar (Supplementary Image 4). Within *Streptococcus*, by contrast, the major species groups distinguishable by the V4-V5 region were each represented primarily by a single oligotype. In summary, some oligotypes were markers for groups of organisms analogous to species or species groups, whereas other oligotypes provided sub-species level information.

## Discussion

### Individualized oligotype profiles within a common framework in plaque

Our analysis with single-nucleotide resolution of the high-quality Jiang et al. time-series data set showed a common framework of plaque-typical genera and species in each individual, but individual-specific microbiota within this framework. At the oligotype level, individuals were almost entirely distinct from one another, despite the fluctuations within each individual over time. Although the species-level composition of the plaque microbiota is very different from the microbiota inhabiting tongue and saliva (Aas et al., 2005; Eren et al., 2014), nonetheless our time-series results with plaque are in broad agreement with previous high-throughput time-series studies of these other oral sites. Our finding that individuals are more similar to themselves over time than they are to other individuals agrees with the conclusions of Costello et al. (2009), Caporaso et al. (2011), Stahringer et al. (2012) and Cameron et al. (2015) for saliva and tongue. Our demonstration of variability within an individual agrees with the findings of Caporaso et al. (2011), David et al. (2014), Ding and Schloss (2014) and Flores et al. (2014). Thus, our results on plaque are broadly consistent with results on microbial dynamics at other sites in that qualities of both stability and variability are displayed. However, how are these apparently paradoxical qualities to be reconciled?

Looking at the community composition underlying the summary distance metrics, our results showed an oligotype-level “fingerprint” characteristic of each individual, consisting of a set of persistently abundant oligotypes with abundance fluctuating around a stable mean over time. The fluctuation around a stable mean resembles the “stationary dynamics” described by David et al. (2014) for the gut and salivary microbiota of two individuals sampled daily over the course of a year. The drivers of the fluctuations remain unexplained, as does the basis for individual distinctiveness of the overall oligotype composition of plaque.

Clues to the physiological or ecological reasons for both similarity and distinctiveness, however, may be found by considering these time series results in the context of the spatial structure of dental plaque. Recently, we demonstrated the consistent presence in plaque of a “hedgehog” structure, a multi-genus microbial consortium that forms around filamentous corynebacteria (Mark Welch et al., 2016). Interestingly, the taxa that we report here to have relatively constant abundance both within and between individuals—*Corynebacterium, Capnocytophaga, Fusobacterium, Actinomyces*, and *Streptococcus*—are among the major participants in this consortium. *Corynebacterium* forms bush-like (or spiny, hedgehog-like) clusters of filaments, providing the structural framework for the consortium. *Streptococcus* binds in abundance to the distal ends of these filaments, forming an outer shell of “corncob” structures. We hypothesize that this shell of *Streptococcus* alters the local biochemical environment by consuming oxygen and secreting lactate, acetate, carbon dioxide, and peroxide (Ramsey et al., 2011; Zhu and Kreth, 2012). Carbon dioxide-loving taxa such as *Capnocytophaga* and anaerobes or micro-aerophiles such as *Fusobacterium* (Diaz et al., 2000) thrive in positions within the structure that match their metabolic requirements, while *Actinomyces* is frequently found adjacent to the hedgehog structure or intermingled with the *Corynebacterium* filaments at its base. The relatively constant abundance of these taxa in the time series data reported here suggests that there is a limit to the individuality of plaque, in that the consortium taxa are consistently present across individuals.

By contrast, the taxa that were among the more variable in the time series data—*Neisseria* and *Prevotella*—are sporadic participants in the hedgehog structure or are absent from it. Members of the family *Neisseriaceae* were detected sporadically in the hedgehog structure, in or near the aerobic outer shell, while *Prevotella* was generally absent from the hedgehog (Mark Welch et al., 2016). The inter-individual variability of these taxa shown here suggests either functional interchangeability of taxa or different biology of plaque in different individuals. For example, the low abundance of *Streptococcus* in the individuals with unusually high *Neisseria* suggests that in these individuals *Neisseria*, an aerobe, may fill a functional role generally carried out by the facultatively aerobic *Streptococcus*. The wide variation in abundance of the obligate anaerobe *Prevotella*, by contrast, may suggest a difference in plaque physiology between individuals with high-*Prevotella* or low-*Prevotella* communities. Whether such differences might result from host-specific factors (Flores et al., 2014) or chance historical events perpetuated within each host by priority effects is an important topic for further investigation.

### Significance of plaque microbiota fingerprints

The physiological or ecological meaning of distinct oligotypes and the overall microbiota fingerprint is likewise an important topic for future study. In our analysis, a 336-nucleotide stretch of the rRNA gene, analyzed with single-nucleotide resolution, acted as a tag for the underlying organism. In the absence of detailed knowledge of the organisms under study, it is difficult to assess how much ecological meaning to assign to these different tags. However, evidence accumulated over several decades indicates that for some groups of organisms, very small differences in the rRNA sequence represent significant evolutionary distances and divergent ecology. Among the enterobacteria, for example, *E. coli* and several species of *Shigella* and *Salmonella* have 16S rRNA gene sequences that are more than 99% identical (Cilia et al., 1996; Fukushima et al., 2002). The same is true of a number of species within the genus *Bacillus* (Ash et al., 1991; Fox et al., 1992) and, within the oral microbiome, the same is true of the abundant commensal *Streptococcus mitis* and the highly pathogenic *S. pneumoniae* (Denapaite et al., 2010; Kilian et al., 2014). Indeed, enormously important differences in biology and pathogenicity can also occur between strains that are considered members of the same species and have identical or nearly-identical 16S rRNA gene sequences (Böddinghaus et al., 1990; Perna et al., 2001; Jin et al., 2002). These findings suggest that even a single nucleotide difference in the 16S rRNA gene can indicate the presence of significant underlying differences in the genomes and functional roles of organisms.

Alternatively, it is also possible that the different versions of the 16S rRNA gene sequence simply represent population-level variation at a neutral site, and that the organisms possessing one or another of these variants are not physiologically different. The data we present here argue against this possibility. If the organisms represented by these sequences were functionally equivalent, they would be expected to vary in relative abundance in a random walk. The pool of available oligotypes is widely shared; most oligotypes in this analysis were detectable, albeit in low abundance, in most individuals. Yet, in most cases, a random walk did not occur; instead, different oligotypes dominated consistently in different individuals. Thus, the stable oligotype profile within each individual suggests that plaque oligotypes indicate the presence not of neutral variants but of evolutionarily selected, ecologically distinct organisms.

The relationship of oligotypes to species-level groupings is not straightforward. Species-level taxonomy itself is not static, but is continually subject to refinement. *Corynebacterium matruchotii*, for example, is thought to contain cryptic species (Barrett et al., 2001). Nonetheless, species groupings represent current knowledge of the biology of the organisms and can provide meaningful insight. Our analysis revealed some cases in which oligotypes apparently correspond to species-level groups or small clusters of described species, such as within the genus *Streptococcus*. If distinct strains were present with differing gene content and physiology but identical 16S rRNA gene sequences, they were, naturally, indistinguishable by this analysis. In other genera, however, the resolving power of the sequenced region of the 16S rRNA gene is greater (or the scrutiny of the genus by microbiologists has been lesser) and oligotypes identified sub-species-level groups, such as within the genera *Corynebacterium* and *Capnocytophaga*.

### Cross-cultural and genus-level consistency with individual variations

Our comparison of plaque 16S rRNA gene sequencing data from China and the United States showed that the plaque microbial community contains the same major genera and spans a similar range of variation in individuals from both cultures. Our results suggest that any systematic differences between Chinese and Western plaque, should they exist, lie in shifts of species composition within abundant genera, or in the presence and abundance of rare genera. In contrast to the lack of ethnic differences we found in the supragingival plaque community, previous studies have reported ethnic distinctions in other oral microbiomes. Mason et al. (2013) studied the saliva and supra- and sub-gingival plaque from four ethnic groups living in America (Chinese, Latino, non-Hispanic whites, and non-Hispanic African Americans) and found significant clustering by ethnicity in sub-gingival and saliva samples but not in supra-gingival plaque. Li et al. (2014) sampled saliva from Africans, Germans, and native Alaskans and found that the African samples differed from the Alaskan and Germans ones by a number of measures, including a high abundance of the genus *Enterobacter*. Takeshita et al. (2014) compared the salivary microbiomes of Koreans and Japanese and found *Neisseria* to be significantly more abundant in Koreans and *Prevotella, Fusobacterium*, and *Veillonella* more abundant in Japanese. A study of the oral mucosa of uncontacted Amerindians showed similar overall diversity to the oral microbiomes of developed Americans, but higher proportions of certain taxa including *Prevotella, Fusobacterium*, and *Gemella* (Clemente et al., 2015). Park et al. (2015) reported an unusual taxon, the halophilic gamma proteobacterium *Halomonas hamiltonii*, to be abundant in subgingival plaque from healthy Korean volunteers. It may be that the presence of ethnic or cultural signatures varies by oral site with certain locations such as saliva and subgingival plaque being more sensitive to ethnic differences than others. Any such signatures, however, were undetectable in this study of the healthy supragingival plaque community.

Regardless of the question of ethnic signatures, our application of oligotype analysis to supragingival plaque highlights the importance of single-nucleotide analysis of microbial communities. Understanding of the individuality, stability and variability, the habitat and community dynamics, and the physiological or ecological meaning of microbial communities all would be deepened by analysis of sequence data at the highest level of resolution possible.

## Author contributions

JMW and GB conceived and designed the work; DU and JMW analyzed the data; and DU, JMW, and GB wrote the paper.

### Conflict of interest statement

The authors declare that the research was conducted in the absence of any commercial or financial relationships that could be construed as a potential conflict of interest.
