# Supplementary material for: Individuality, Stability, and Variability of the Plaque Microbiome
Source: Front Microbiol. 2016 Apr 22;7:564. doi: 10.3389/fmicb.2016.00564 (PMC4840391; doi:10.3389/fmicb.2016.00564)
Supplement: Supplementary file 2 [file DataSheet2.PDF]

Supplementary Data Sheet 2

Individuality, stability, and variability of the plaque microbiome

Daniel R. Utter*, Jessica L. Mark Welch, Gary G. Borisy

*** Correspondence:** Daniel R. Utter, Department of Organismic and Evolutionary Biology, Harvard University, Cambridge, MA, 02138, USA

dutter@g.harvard.edu

Assessing possible sources of error in classification.

Considering that *Actinomyces* and *Parascardovia* share the same phylum, we hypothesized that sequences representing *Actinomyces* were erroneously classified as *Parascardovia* in Jiang et al.'s original analysis. In an effort to understand the source of the misclassification, we used NCBI's BLAST blastn program (www.ncbi.nlm.nih.gov) to query the entire data set of 359,565 raw, untrimmed reads against the SILVA database (SILVA 106; www.arb-silva.de), following the taxon-assignment methodology described in Jiang et al. (2015). Through this process we identified only six reads for which a match to *Parascardovia* was reported within the top 50 hits, of which the best *Parascardovia* match was 97% identity. Even for these six reads BLAST also reported a better match to a *Scardovia* sequence, as judged by bit score for all 6 sequences and as judged by percent identity for five out of the six sequences. We therefore conclude that there were very few or no legitimate *Parascardovia* reads in the dataset.

Another potential source of misclassification could have been the presence of sequences representing both *Actinomyces* and a *Parascardovia*-like taxon in the same OTU, which then came to be labeled as *Parascardovia*. We investigated whether the reference sequences for *Actinomyces* and *Parascardovia* were within 94% of one another. We used TaxMan (Brandt et al., 2012; www.ibi.vu.nl/programs/taxmanwww/) to trim the SILVA 108 database to the region targeted by the primers used in Jiang et al. (2015) and generated a distance matrix for these sequences using o-sequence-distances (part of the oligotyping pipeline, available at github.com/meren/oligotyping). We found no *Parascardovia* reference sequences with above 90% similarity to any *Actinomyces* reference sequences in the SILVA 108 database (Supplementary Image 2). Thus, misclassification could not be accounted for by similarity of *Actinomyces* and *Parascardovia*. We were unable to identify the exact source of the misclassification.
