# Supplementary material for: Individuality, Stability, and Variability of the Plaque Microbiome
Source: Front Microbiol. 2016 Apr 22;7:564. doi: 10.3389/fmicb.2016.00564 (PMC4840391; doi:10.3389/fmicb.2016.00564)
Supplement: Supplementary file 4 [file Image1.PDF]

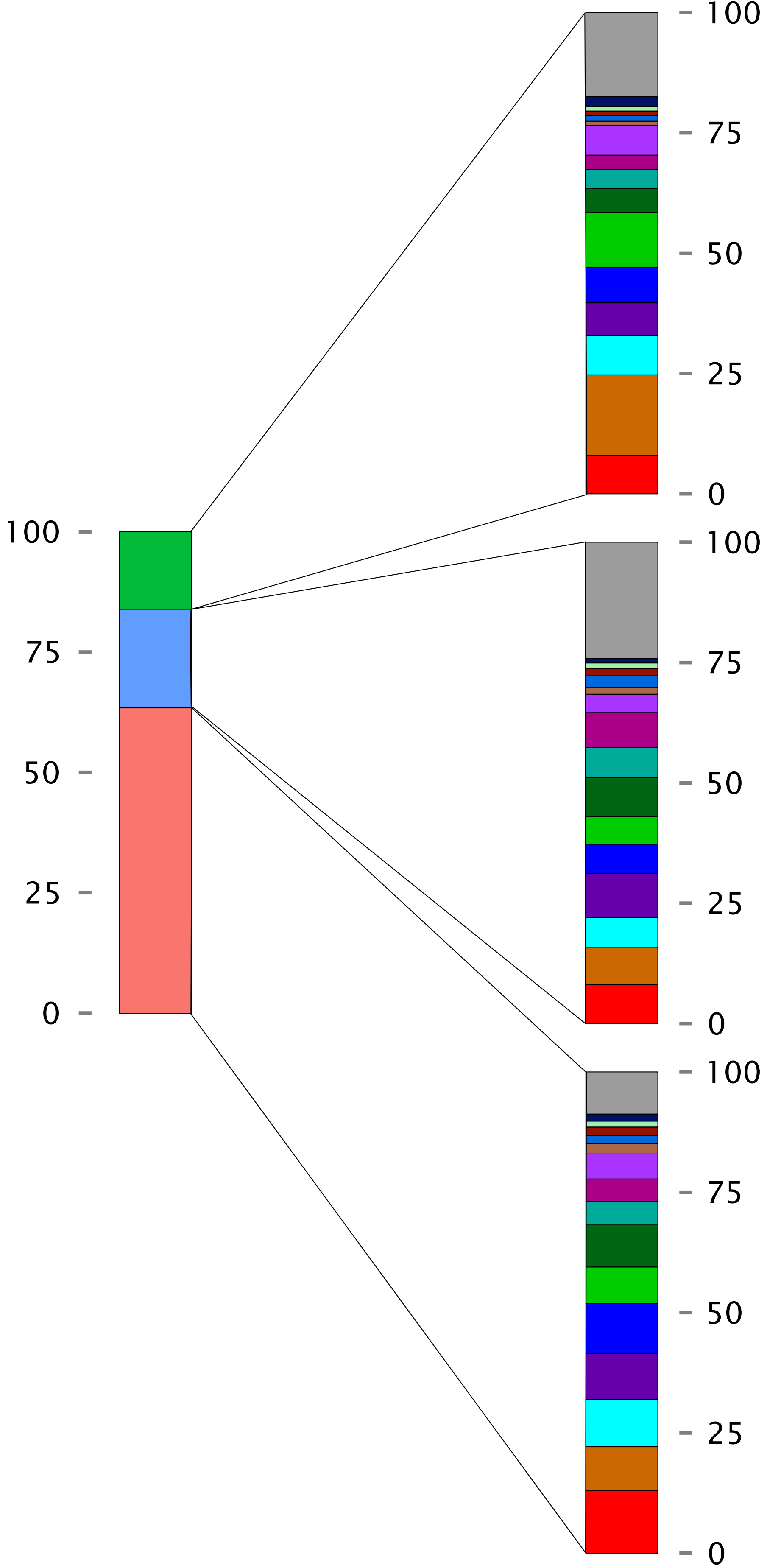

Supplementary Image 1. Reads eliminated from the analysis have substantially the same taxonomic distribution as reads kept. The left stackbar shows the percentages of the entire data set (359,565 reads) that were kept (pink), removed during MED (blue), or removed during the trimming/alignment process (green). For each group in the left stackbar, the corresponding stackbar on the right shows the relative abundance, by using GAST against the HOMD RefSeq v.13.2 database (Dewhirst et al., 2010), of the same 15 genera as shown in Figure 1D.
