# Supplementary material for: Individuality, Stability, and Variability of the Plaque Microbiome
Source: Front Microbiol. 2016 Apr 22;7:564. doi: 10.3389/fmicb.2016.00564 (PMC4840391; doi:10.3389/fmicb.2016.00564)
Supplement: Supplementary file 5 [file Image2.PDF]

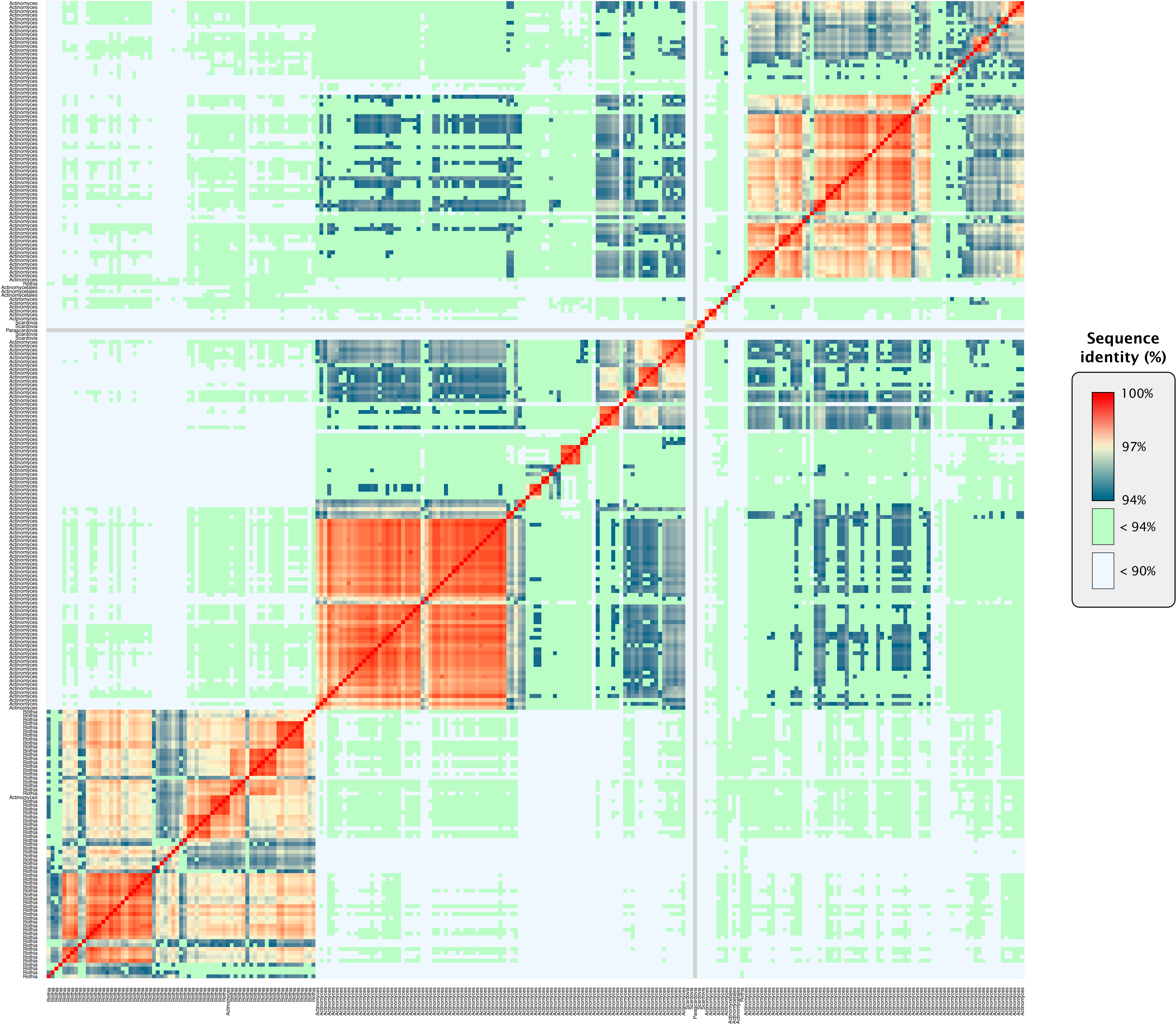

Supplementary Image 2. Sequence similarity between SILVA 108 Scardovia, Parascardovia, Rothia, Actinomyces, and Actinomycetales reference sequences in the V4-V5 region. Shown is a heatmap of sequence similarity in the V4V5 region for reference sequences belonging to the genera *Scardovia*, *Parascardovia*, *Rothia*, *Actinomyces*, and *Actinomycetales* in the SILVA 108 database. Grey boxes represent sequence comparisons involving Parascardovia with less than 90% sequence identity.
