# Supplementary material for: Individuality, Stability, and Variability of the Plaque Microbiome
Source: Front Microbiol. 2016 Apr 22;7:564. doi: 10.3389/fmicb.2016.00564 (PMC4840391; doi:10.3389/fmicb.2016.00564)
Supplement: Supplementary file 6 [file Image3.PDF]

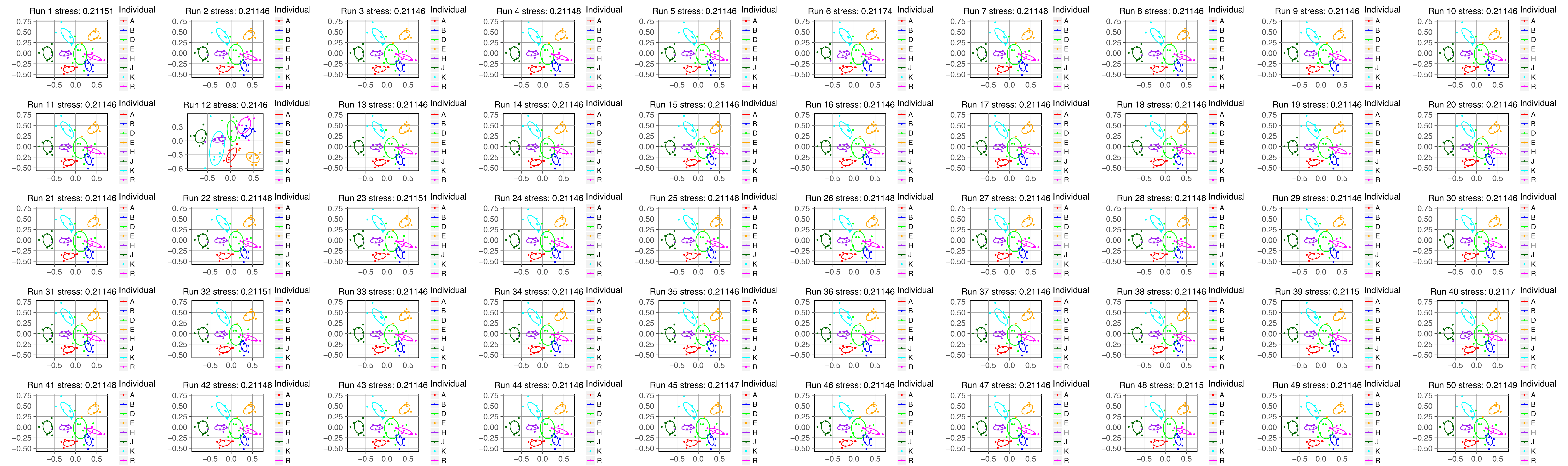

Supplementary Image 3. Individuals can be distinguished at the oligotype level. .This image shows the results of repeating the MDS analysis shown in Figure 3D 50 independent times. 49 of the 50 MDS analyses show the same topology as that shown in Figure 3D. Run 12 shows the alternate topology.
