# Supplementary material for: Individuality, Stability, and Variability of the Plaque Microbiome
Source: Front Microbiol. 2016 Apr 22;7:564. doi: 10.3389/fmicb.2016.00564 (PMC4840391; doi:10.3389/fmicb.2016.00564)
Supplement: Supplementary file 7 [file Image4.PDF]

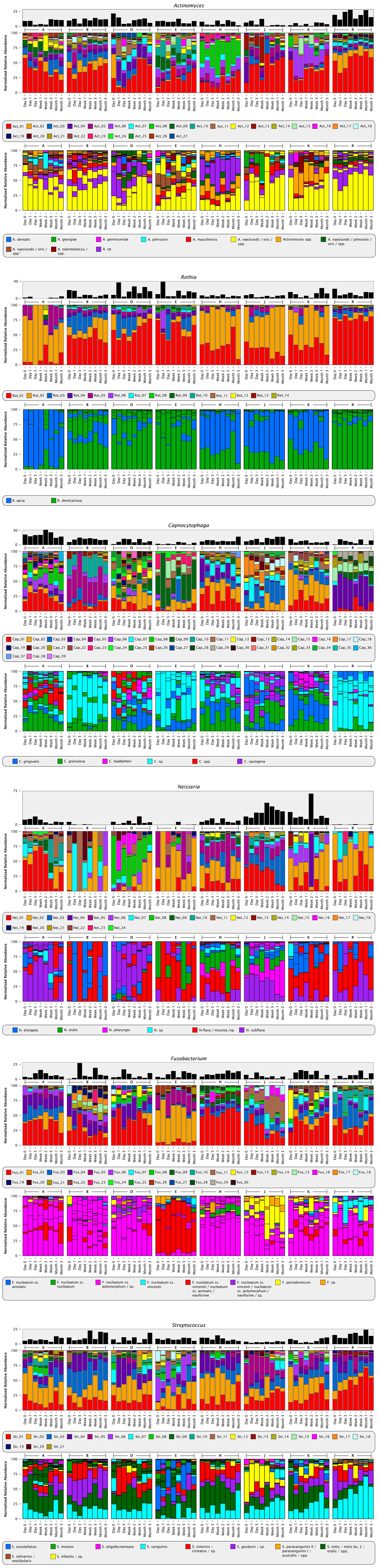

Supplementary Image 4. Relative oligotype compositions of Actinomyces, Rothia, Capnocytophaga, Neisseria, Fusobacterium, and Streptococcus in each sample. The smaller, black-and-grey bar above each larger, colored stackbar shows the relative abundance of the genus in each sample; the colored bar directly underneath shows the normalized relative abundance of each oligotype observed in that sample. The oligotype ID shown in each legend is an abbreviated reference to the oligotype ID in Supplemental Data S2, e.g. "Act_01" refers to oligotype ID "Actinomyces_01". Abbreviations are "Act" for "Actinomyces"; "Rot" for "Rothia"; "Cap" for "Capnocytophaga"; "Nei" for "Neisseria"; "Fus" for "Fusobacterium"; "Str" for "Streptococcus." The bottom stackbar, representing species designations for oligotypes, is arranged the same as the oligotype stackbar above, but blocks are colored by species or species group rather than by oligotype. For oligotypes equally similar to reference sequences from two or more species, all matched species are presented as a species group.
