# Supplementary material for: Individuality, Stability, and Variability of the Plaque Microbiome
Source: Front Microbiol. 2016 Apr 22;7:564. doi: 10.3389/fmicb.2016.00564 (PMC4840391; doi:10.3389/fmicb.2016.00564)
Supplement: Supplementary file 8 [file Image5.PDF]

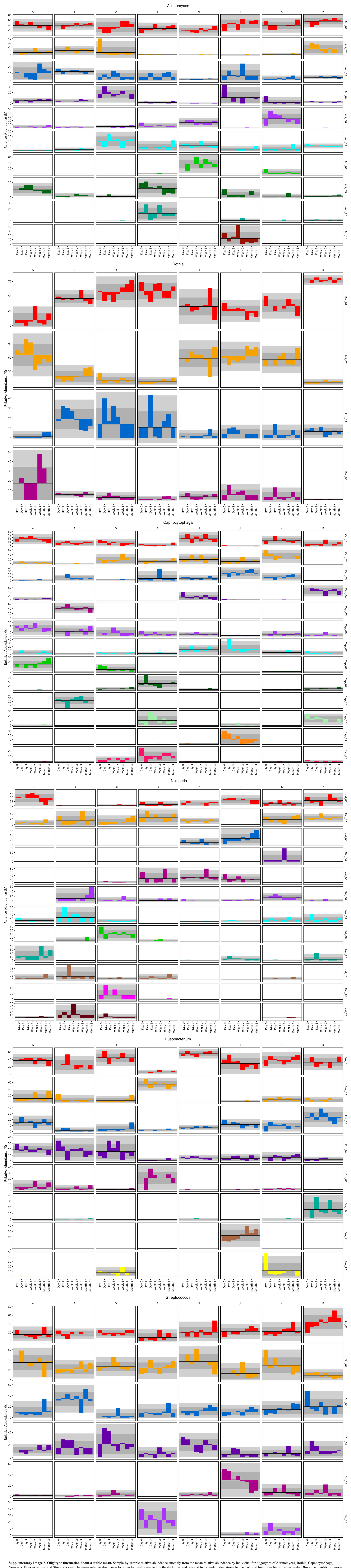

Supplementary Image 5. Oligotype fluctuation about a stable mean. Sample-by-sample relative abundance anomaly from the mean relative abundance by individual for oligotypes of Actinomyces, Rothia, Capnocytophaga, Neisseria, Fusobacterium, and Streptococcus. The mean relative abundance for an individual is marked by the dark line, and one and two standard deviations by the dark and light grey fields, respectively. Oligotype identity is denoted on the right side of each row.
